# Linear Friction Welding of an AZ91 Magnesium Alloy and the Effect of Ca Additions on the Weld Characteristics

**DOI:** 10.3390/ma14113130

**Published:** 2021-06-07

**Authors:** Luis Angel Villegas-Armenta, Priti Wanjara, Javad Gholipour, Isao Nakatsugawa, Yasumasa Chino, Mihriban Pekguleryuz

**Affiliations:** 1 Department of Mining and Materials Engineering, McGill University, 3610 University Street, Montreal, QC H3A 2B2, Canada; mihriban.pekguleryuz@mcgill.ca; 2 National Research Council Canada, Aerospace Research Center, 2107 Chemin de la Polytechnique, Campus de l’Université de Montréal, Montréal, QC H3T 1J4, Canada; priti.wanjara@cnrc-nrc.gc.ca (P.W.); javad.gholipourbaradari@cnrc-nrc.gc.ca (J.G.); 3 Multi-Material Research Institute, National Institute of Advanced Industrial Science and Technology, 2266-98, Anagahora, Shimoshidami, Moriyama-ku, Nagoya, Aichi 463-8560, Japan; i.nakatsugawa@aist.go.jp (I.N.); y-chino@aist.go.jp (Y.C.)

**Keywords:** magnesium, calcium, linear friction welding, aerospace, texture, X-ray diffraction, tensile testing, digital image correlation, strain mapping, FactSage-FTlite

## Abstract

Solid-state welding offers distinct advantages for joining reactive materials, such as magnesium (Mg) and its alloys. This study investigates the effect of linear friction welding (LFW) on the microstructure and mechanical properties of cast AZ91 (Mg–9Al–1Zn) and AZ91–2Ca alloys, which (to the best knowledge of the authors) has not been reported in the literature. Using the same set of LFW process parameters, similar alloy joints—namely, AZ91/AZ91 and AZ91–2Ca/AZ91–2Ca—were manufactured and found to exhibit integral bonding at the interface without defects, such as porosity, inclusions, and/or cracking. Microstructural examination of the AZ91/AZ91 joint revealed dissolution of the Al-rich second phase in the weld zone, while the Mn containing phases remained and were refined. In the AZ91–2Ca/AZ91–2Ca joint, the weld zone retained Ca- and Mn-rich phases, which were also refined due to the LFW process. In both joint types, extensive recrystallization occurred during LFW, as evidenced by the refinement of the grains from ~1000 µm in the base materials to roughly 2–6 µm in the weld zone. These microstructural changes in the AZ91/AZ91 and AZ91–2Ca/AZ91–2Ca joints increased the hardness in the weld zone by 32%. The use of digital image correlation for strain mapping along the sample gage length during tensile testing revealed that the local strains were about 50% lower in the weld zone relative to the AZ91 and AZ91–2Ca base materials. This points to the higher strength of the weld zone in the AZ91/AZ91 and AZ91–2Ca/AZ91–2Ca joints due to the fine grain size, second phase refinement, and strong basal texture. Final fracture during tensile loading of both joints occurred in the base materials.

## 1. Introduction

Weight saving in commercial aircraft [1] is one of the strategic routes to advance the target of near-zero or zero CO_2_ emissions to combat climate change [2]. When designing new components, the use of lower density materials can provide additional weight savings when combined with structurally efficient designs [3]. Magnesium (Mg) is one of the lightest structural metals and, with a density (ρ) of 1.7 g/cm^3^, it provides a significant advantage over other aerospace materials, such as aluminum (ρ = 2.7 g/cm^3^) or titanium (ρ = 4.5 g/cm^3^). Certain drawbacks, such as the perceived ignition risk, have traditionally hindered the widespread use of Mg in commercial aircraft. Solid Mg reacts readily with oxygen, forming a magnesium oxide (MgO) scale that is fairly compact and protective up to 475 °C [4]. Above this temperature, the accelerated oxidation of Mg increases the surface temperature and promotes Mg evaporation due to its intrinsic high vapor pressure (low boiling temperature of 1091 °C) [5] that eventually leads to ignition, once the liquid state is reached.

It is known that the ignition tendency of Mg is composition dependent. Mg alloys with alkaline earth [6,7] and rare-earth elements [8,9,10] acquire ignition resistance through the formation of a protective oxide scale that hinders interaction of oxygen with molten Mg. Calcium (Ca) additions have been observed to increase the ignition resistance of Mg–Al–Zn (AZ) alloys [11,12,13]. Ca also improves other properties of Mg alloys, such as corrosion resistance and creep strength [14,15,16]. On the other hand, the elongation and tensile strength may decrease when higher levels of Ca are added [17].

Mg alloy castings have a great potential to increase the buy-to-fly ratio of aerospace components close to 1 (i.e., having a net-shape geometry that generates minimal material waste during production). Moreover, the buy-to-flight ratio of aerospace assemblies can also be minimized using welding techniques instead of mechanical fastening [18]. However, due to the reactivity of Mg and its alloys, most fusion welding processes require shielding of the molten metal using protective gases, such as argon (Ar) or helium (He) [19], so as to prevent oxidation and, in turn, oxide inclusions in the weld. Even so, the welding process is complicated by significant evaporation of Mg from the molten pool that weakens the weld regions [20]. Furthermore, during fusion welding, Mg alloys are susceptible to liquation and solidification cracking [21], as well as to grain growth in the heat-affected zone that has been reported to lower the mechanical performance of the joint [22]. Thus, there are significant challenges for developing stable and high-throughput welding conditions, while controlling the weld quality and microstructure in Mg alloy joints; this is exacerbated for alloy compositions with Ca additions that are inherently brittle.

**Figure 1:**
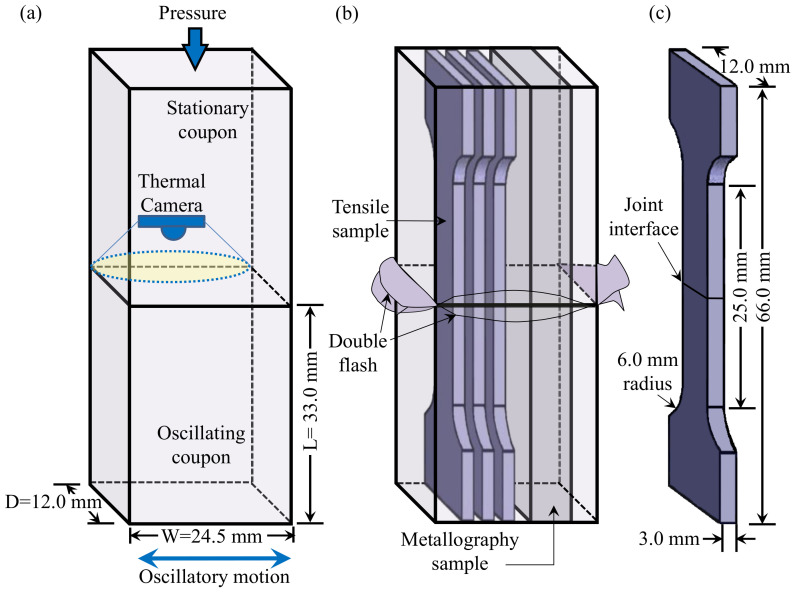
Schematic diagram showing (**a**) the LFW coupon length (L), width (W), and depth (D); (**b**) the machining plan for extracting the metallography and tensile samples; and (**c**) the geometry of the tensile samples.

To address these challenges, solid-state joining techniques, such as friction stir welding (FSW), have been proposed to join Mg alloys [23,24,25]. During FSW, a non-consumable rotating tool is applied at the joint interface between the workpieces to generate the frictional heat required to soften (plasticize) the metal and enable adequate mixing for solid-state bonding. However, the nature of this technique limits its use mostly to sheet and plate forms; bulky workpieces or complex geometries obtained through casting techniques cannot be effectively welded by FSW. For such geometries, linear friction welding (LFW) becomes an important alternative. This process consists of joining two surfaces through the frictional heat generated by displacing one workpiece against the other (Figure 1a). The movement is done in a reciprocating manner, at high frequencies and by applying a pressure perpendicular to the displacement direction [26]. This solid-state joining technique is especially well suited for difficult-to-weld reactive materials that experience melting and solidification issues [27]. However, in the case of similar Mg alloy welds, at the time of writing, there is no reported work that describes the influence of the LFW process on the microstructure and mechanical properties of the joints. The closest is a study by Bhamji et al. [28] on LFW of dissimilar aluminum alloy (AA) 6082-T6 to AZ31 Mg alloy; their research indicated that the remnant pores and intermetallic compounds formed at the weld interface of the dissimilar welds limit the mechanical performance of the joints. Apart from these findings, the microstructural transformations occurring in the AZ31 Mg alloy due to the LFW process were not examined in their study [28].

Considering this, the present work was undertaken to comprehensively study the effectiveness of LFW for two cast Mg alloys of importance to the aerospace and automotive industries: Mg–9Al–1Zn–0.3Mn (AZ91) and AZ91 + 2 wt.% Ca (AZ91–2Ca). It is noteworthy that Ca was selected as a compositional addition in this study due to its positive effect in increasing the ignition resistance of AZ91, as well as improving the corrosion [29,30,31,32] and high-temperature oxidation [33,34] resistance. These characteristics are of particular importance for aerospace application, and therefore, this addition was made. Thus, two types of similar alloy welds were examined in this study: AZ91 to AZ91 (hereafter, AZ91/AZ91) and AZ91–2Ca to AZ91–2Ca (hereafter. AZ91–2Ca/AZ91–2Ca). During LFW, the thermal conditions at the joint surfaces on the AZ91/AZ91 and AZ91–2Ca/AZ91–2Ca welds were evaluated to support understanding of the interfacial characteristics and microstructural transformations (texture and phase constituents) that were observed through microscopic and X-ray diffraction (XRD) techniques, as well as calculated using FactSage 8.0 and FTlite (2020), a database for thermodynamic and phase equilibria data. In addition, the performance of the AZ91/AZ91 and AZ91–2Ca/AZ91–2Ca welds was assessed through two-dimensional hardness mapping across the joints, as well as tensile testing with digital image correlation for mapping the strain distribution in the weld zone relative to the base material regions.

## 2. Materials and Methods

The AZ91–2Ca alloy, used in this study, was synthesized in McGill’s Light Metals Research Laboratory (Montréal, Québec, Canada). The alloy was melted using a Norax Induction Furnace (20 kW/5 kHz) (Levis, QC, Canada) using a graphite crucible under a protective SF_6_/CO_2_ atmosphere. The pure Mg used to synthesize the alloys was supplied by Magnesium Elektron Limited (Swinton, Manchester, UK) with a purity of 99.9%, while the Mg-30 wt.% Ca and Mg-0.75 wt.% Mn master alloys were provided by Timminco Metals (Toronto, ON, Canada). The pure Al and Zn pellets, used as additions, were supplied by Alfa Aesar (Tewksbury, MA, USA). First, pure Mg was melted at 690 °C; then, the Mg–Mn master alloy, Mg–Ca master alloy, and pure elements were added consecutively in that order. The molten alloy was stirred after each addition using a graphite rod to homogenize the melt; then, the alloy was held for 10 min to ensure complete dissolution, followed by pouring at 730 °C into an H13 tool-steel mold preheated at 400 °C and coated with boron nitride to assist casting ejection. The resulting cast plates were 12.5 cm × 10 cm with a thickness of 2.5 cm. The composition of the AZ91–2Ca alloy is presented in Table 1. This was determined by a NADCAP-certified laboratory, Genitest Inc. (Montréal, Québec, Canada), using inductively coupled plasma-atomic emission spectrometry (ICP-AES). For the AZ91 alloy, a commercial alloy ingot was used. Its chemical composition is given in Table 1.

Coupons for LFW—12.0 mm (D) × 24.5 mm (W) × 33.0 mm (L)—were machined with a tolerance of 0.02 mm from the cast plates of AZ91 and AZ91–2Ca. The faying surfaces of the coupons were lightly sanded at the joint interface using 320-grit sandpaper and then cleaned with ethanol just before placing them in the LFW sample holder. The equipment used for LFW was an MTS LFW process development system (PDS) at the National Research Council Canada’s Aerospace Manufacturing Technology Center (Montréal, QC, Canada), which consisted of two hydraulic actuators: an in-plane actuator oscillating the lower coupon in the horizontal direction and a forge actuator applying a downward load through the top stationary coupon. More details about the technical specifications of the MTS LFW PDS system and the LFW process are provided in [36].

The LFW experiments were conducted in air (without shielding gas protection) at an ambient temperature of 22 °C using a pressure of 50 MPa, a frequency of 50 Hz, and an oscillating amplitude of 2 mm. During LFW, temperature measurements were made using a FLIR SC8300HD thermal camera (Wilsonville, OR, USA). To assure the accuracy of the surface temperature measurements, the thermal camera used in this study was blackbody calibrated to NIST traceability. However, having calibrated the thermal camera on a blackbody source (or perfect radiator), the ability to measure the temperature on the interface of the AZ91/AZ91 and AZ91–2Ca/AZ91–2Ca joints depends directly on the actual surface emissivity within the process temperature range. Hence, the methodology used to determine the average emissivity value involved heating the AZ91 and AZ91–2Ca coupons to 500 °C in a furnace and monitoring their actual temperature using a thermocouple attached to their surface. Then, the surface temperatures on the AZ91 and AZ91–2Ca coupons were also measured with the thermal camera. The emissivity was adjusted by matching the surface temperature measurements from the thermal camera to that of the contact thermocouple during cooling from 500 to 250 °C. This resulted in an average emissivity of 0.58.

After LFW, metallographic and tensile test samples were extracted from the AZ91/AZ91 and AZ91–2Ca/AZ91–2Ca welds, as illustrated in Figure 1b. Metallographic preparation of the transverse weld samples involved mechanical grinding with 1200 grit SiC paper and water to render the surface planar, followed by final polishing with a 0.05 µm colloidal silica suspension on an Anamet Imperial™ polishing cloth (Frankford, Ontario, Canada). The microstructural characteristics of the welds were examined on a polished surface using backscattered electron (BSE) imaging at an accelerating voltage of 15 kV on a SU3500 Hitachi scanning electron microscope (SEM) (Etobicoke, Ontario, Canada). This microscope was also equipped with an energy-dispersive X-ray spectroscopy (EDS) detector for elemental analysis/mapping and an electron backscattered diffraction (EBSD) detector to analyze the micro-texture of the base materials and welds. Additionally, an XRD was used to determine the intermetallic compounds in both the base materials and the weld region using a voltage of 40 kV with a scan step time of 300 s. The samples were placed inside a Bruker X-ray diffractometer (Vancouver, British Columbia, Canada) equipped with a Cu Kα radiation source of λ = 1.54060 Å, making a scan between 20° and 90° for 2 θ.

Vickers microhardness testing across transverse weld sections (i.e., perpendicular to the oscillation direction) was performed on polished (mirror finished) surfaces of the metallography samples using a Struers DuraScan80 hardness tester (Ballerup, Denmark) equipped with a motorized x-y stage and a built-in microprocessor for automated measurement of hardness values. Hardness profiles were determined based on the average of three measurements for each point with a load of 500 g and a dwell period of 15 s at an indent spacing of 0.2 mm. The minimum test point separation distance for all measurements was at least three times the diagonal measurement of the indent to avoid any potential effect of strain fields from the neighboring indents.

Guided by the principles given in ASTM E8M-16a [37], standard sub-size tensile samples were extracted from the linear friction welds and machined to the geometry shown in Figure 1c. The tensile samples were tested at room temperature using a 250 kN MTS testing frame (Eden Prairie, MN, USA) integrated with a laser extensometer and a noncontact optical 3D deformation measurement system (referred often as digital image correlation), Aramis^®^ (GOM-Trillion Quality Systems, King of Prussia, PA, USA). Prior to testing, one side of the tensile sample was marked with two pieces of retroreflective tape to define the gage length for the laser extensometer measurements during testing. On the opposite side, the surface of the tensile sample was first painted with a white background and then a high-contrast random pattern of black speckles was applied. As the functionality of the Aramis^®^ system is sensitive to the quality of this speckle pattern, verification of pattern recognition was performed before tensile testing to ensure proper strain recording along the entire gage length, as described in [38,39]. Tensile tests were conducted until rupture using displacement control at a rate of 0.4 mm/min, which corresponds to an average strain rate of 0.015 min^−1^. To obtain the global stress–strain curves and related mechanical properties, the load data collected from the tensile testing machine were used to calculate the engineering stresses during the test, while the related strains were calculated from the displacement obtained from the laser extensometer. The tensile properties evaluated in this work included the yield strength (YS), ultimate tensile strength (UTS), and elongation (EL) for each sample. Three tensile samples for each weld combination were tested to obtain the average stress–strain response.

## 3. Results

### 3.1. Thermal History and Macroscopic Examinations

**Figure 2:**
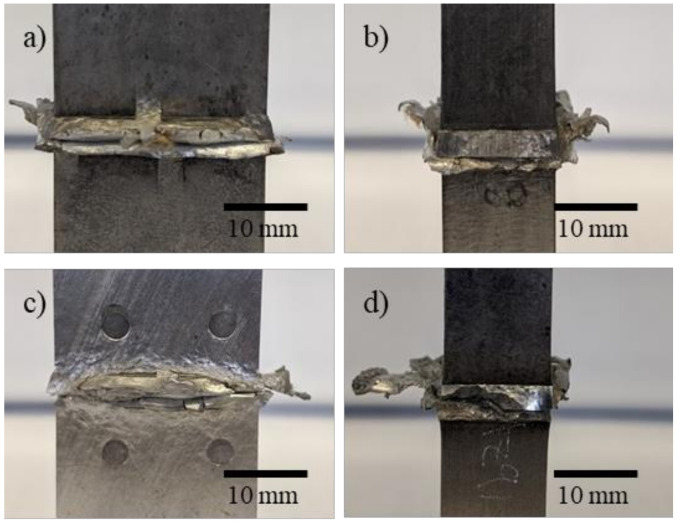
Front and side images of the welded (**a**,**b**) AZ91 and (**c**,**d**) AZ91–2Ca. Both similar Mg alloy welds display discontinuous flash formation.

**Figure 3:**
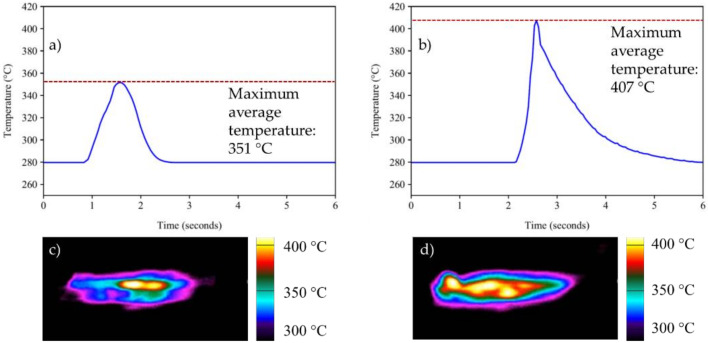
Thermal camera temperature readings at the joint interface and the evolution in the average temperature at the joint interface surface for AZ91 (**a**,**c**) and AZ91–2Ca (**b**,**d**) during LFW.

Figure 2 shows images of the joint interface for the AZ91/AZ91 and AZ91–2Ca/AZ91–2Ca welds after LFW. Both joints exhibited discontinuous flash around the interface region, with some material being extruded/expelled outwards. Thermal camera temperature readings taken during LFW of the AZ91 and AZ91–2Ca alloys were analyzed to plot the evolution of the average temperature at the joint interface with welding time, as shown in Figure 3. In addition, the insets next to each representative curve in Figure 3 for the AZ91 and AZ91–2Ca alloys display a map of the temperature distribution that was captured at a welding time when the maximum temperatures occurred (i.e., light yellow color in the images and scale bar). It is noteworthy that the average temperature was defined from a line crossing the middle of this region. Overall, the average temperature during LFW of AZ91 (351 °C) was lower than that observed for AZ91–2Ca (407 °C).

**Figure 4:**
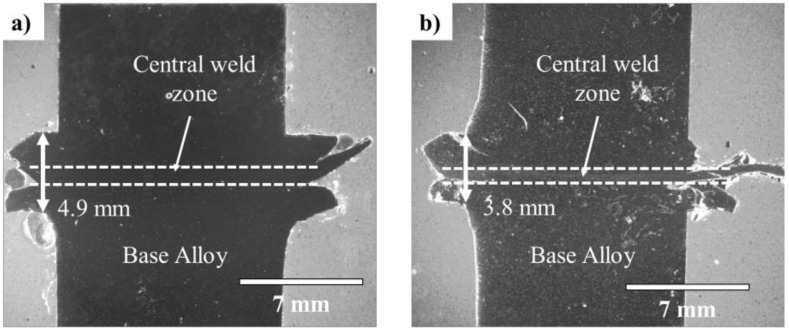
Stereoscopic images of the weld cross-sections (**a**) AZ91/AZ91 and (**b**) AZ91–2Ca/AZ91–2Ca alloys. The dotted lines represent the boundaries of the weld zone.

The stereoscopic images in Figure 4 reveal continuous bonding at the joint line from edge to edge without the presence of any visible porosity, oxides, and/or inclusions. An extruded flash layer was observed for both joints and measured to be thicker in the AZ91/AZ91 weld (4.9 mm) compared to the AZ91–2Ca/AZ91–2Ca weld (3.8 mm). It is worth mentioning that this was measured considering only the portion of material extruded outwards. Within the extruded flash, on the peripheries of AZ91/AZ91 and AZ91–2Ca/AZ91–2Ca welds, there was a remnant weld line or interface between the two sides of the joint (Figure 4). However, within the AZ91/AZ91 and AZ91–2Ca/AZ91–2Ca welds, there were no traces of the prior weld line, which points to the process parameters during LFW being adequate for bringing the faying surfaces into intimate contact to realize sufficient/integral bonding. Instead, within both welds, a central weld zone was evident with a thickness of around 1.5 mm in the AZ91/AZ91 weld and 0.9 mm in the AZ91–2Ca/AZ91–2Ca weld.

### 3.2. Microstructural Characteristics of the Base Materials and Weld Zone Phase Identification

**Figure 5:**
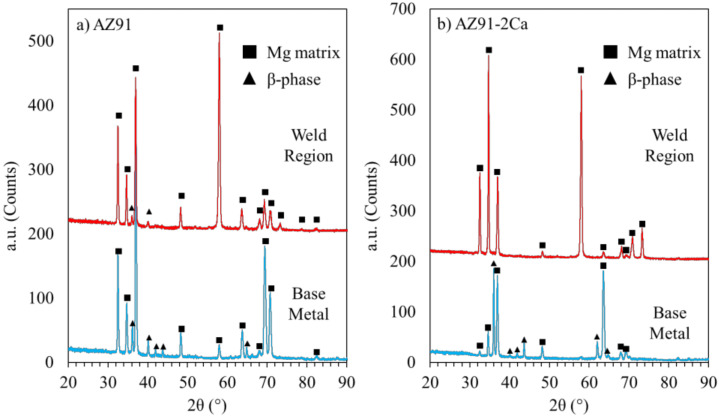
XRD spectra for both the central weld zone and base material in (**a**) AZ91/AZ91 and (**b**) AZ91–2Ca/AZ91–2Ca welds.

Figure 5 shows the XRD spectra of the base materials and the weld zones in the center of the AZ91/AZ91 and AZ91–2Ca/AZ91–2Ca welds. The phases detected were the hexagonal closed-packed (HCP) Mg matrix and the intermetallic β–Mg_17_(Al, Zn)_12_, also known as the β-phase. For the AZ91/AZ91 linear friction weld, both phases were present in the base material and the central weld zone. For AZ91–2Ca/AZ91–2Ca weld, however, no significant peaks belonging to the β–Mg_17_(Al, Zn)_12_ phase were observed in the central weld zone, though they were present in the XRD spectrum of the AZ91–2Ca alloy.

Other phases with a smaller volume fraction may be present, but were not detectable via XRD. FactSage-FTlite™ simulations were performed to predict the additional phases that may form given the composition of the alloys. Scheil–Gulliver calculations were utilized wherein full mixing in the liquid and no diffusion in the solid were considered; this calculation simulated the fast solidification [40] experienced in permanent mold castings. The phases predicted are shown in Table 2.

### 3.3. Microstructural Investigation

**Figure 6:**
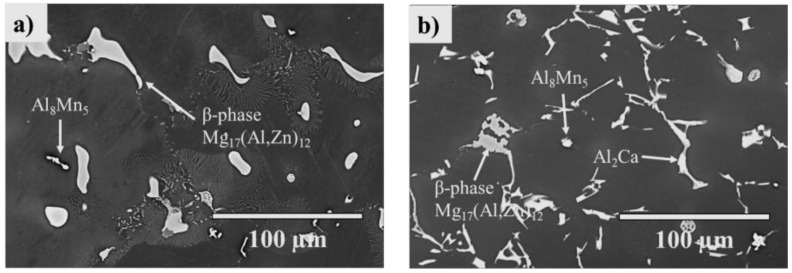
SEM micrographs of the (**a**) AZ91 and (**b**) AZ91–2Ca alloys. Both alloys contain the β–Mg_17_(Al, Zn)_12_ (bulk and lamellar) and Al_8_Mn_5_ phases. The addition of Ca to AZ91 forms the Al_2_Ca phase.

Figure 6a,b shows the SEM micrographs of the cast AZ91 and AZ91–2Ca alloys before LFW. Both Mg alloys contain the second phase β–Mg_17_(Al, Zn)_12_—that was also observed through XRD—along with Mn-rich smaller particles, previously identified as Al_8_Mn_5_ in the literature concerning AZ91 [41]. Additionally, the AZ91–2Ca alloy contained the Al_2_Ca phase in the form of plates connected to the network of the β–Mg_17_(Al, Zn)_12_ phase; previously, the presence of the Al_2_Ca phase has been reported in the literature involving Ca addition to AZ91 [17]. Phase identification for both Mg alloys agrees with the thermodynamic predictions presented in Table 2 with the exception of the Mg_6_(Al, Zn)_5_–φ phase predicted in the AZ91–2Ca alloy that was not observed via SEM. Its composition is similar to the more predominant β–Mg_17_(Al, Zn)_12_ phase; hence it may coexist in small quantities with this phase at the interdendritic regions.

**Figure 7:**
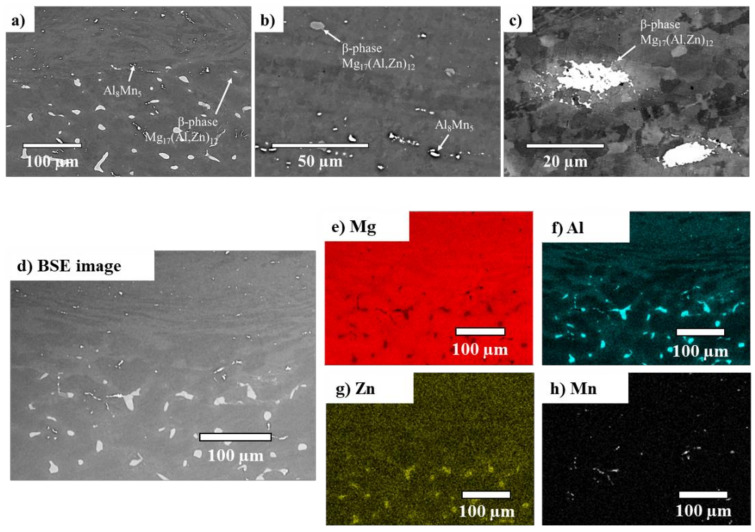
AZ91/AZ91 weld. SEM micrographs of (**a**) the transition between the base material (bottom) and the central weld zone (top), (**b**) the central weld zone, and (**c**) a β–Mg_17_(Al, Zn)_12_ particle located close to the central weld zone. Start of central weld zone in the AZ91/AZ91 weld (**d**) is shown along with its EDS elemental maps (**e**–**h**). Al and Zn concentrated initially at the β–Mg_17_(Al, Zn)_12_ phase, while Mn remained in the Al_8_Mn_5_ phase. After welding, they dissolved into the Mg matrix.

The AZ91/AZ91 weld region consisted of a central weld zone with an abrupt transition to the base material, as shown in Figure 7a. Here, the β–Mg_17_(Al, Zn)_12_ particles became progressively smaller when traversing from the base material (bottom) towards the central weld zone (top). Thus, this central weld zone (Figure 7b) contained a small number of refined β–Mg_17_(Al, Zn)_12_ particles, along with some fragmented Al_8_Mn_5_ stringers that were oriented parallel to the welding direction and remained in the microstructure despite the deformation process. A closer look at the β–Mg_17_(Al, Zn)_12_ particles (Figure 7c) revealed their gradual disappearance, roughly midway into the central weld zone. Figure 7d–h shows the EDS elemental maps of the weld region between the AZ91 base material (bottom) and the central weld zone (top). From the EDS maps, it can be seen that the Al (Figure 7f) and Zn (Figure 7g) distributions also changed; they were concentrated at and in the vicinity of the β–Mg_17_(Al, Zn)_12_ phase in the base material, but were dispersed throughout the matrix in the central weld zone.

**Figure 8:**
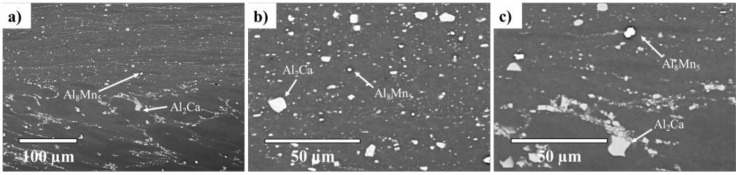
AZ91–2Ca/AZ91–2Ca weld. SEM micrographs of (**a**) the weld region between the base material (bottom) and the central weld zone (top), (**b**) the central weld zone, and (**c**) fragmentation of Al_2_Ca particles close to the central weld zone.

**Figure 9:**
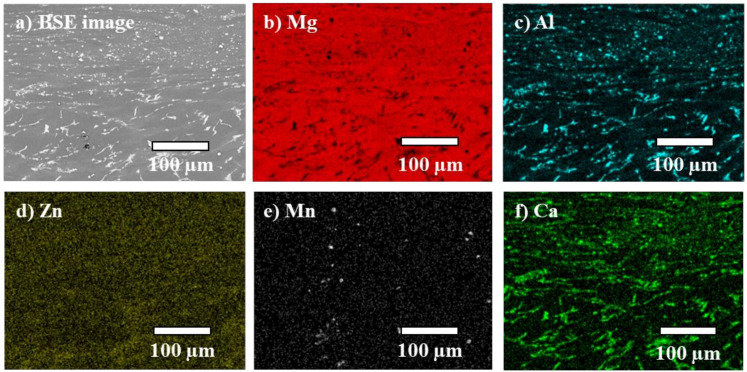
(**a**) AZ91–2Ca/AZ91–2Ca weld. (**b**–**e**) EDS elemental mapping of the weld region between the base material (bottom) and the central weld zone (top). Al (**c**) is seen in the Al_2_Ca particles, while Zn (**d**) appears to be dispersed in the matrix. Mn (**e**) remained in the Al_8_Mn_5_ phase. Towards the weld region, Ca (**f**) predominates in the smaller particles.

Similar to the AZ91/AZ91 weld, the AZ91–2Ca/AZ91–2Ca weld also indicated an abrupt transition between the base material and the central weld zone (Figure 8a). However, in the AZ91–2Ca/AZ91–2Ca weld, the central weld zone contained fragmented Al–Ca particles, in addition to the Al–Mn phase (Figure 8b,c); these are further demonstrated in the EDS maps illustrated in Figure 9. These particles are the Al_2_Ca and the Al_8_Mn_5_ predicted by the FactSage-FTlite™ calculations. Similar microstructural phases and particles have been observed in Ca containing Mg–Al alloys joined via FSW and friction stir processing (FSP), including studies on AZ91–1Ca (Mg–9Al–1Zn–1Ca) [42], AMX602 (Mg–6Al–0.5Mn–2Ca) [43], and AZX612 (Mg–6Al–1Zn–2Ca) [44]. Specifically, the microstructures in these alloys after FSW/FSP displayed a fine dispersion of Al_2_Ca and refined or dissolved β–Mg_17_Al_12_, similar to our observations on the AZ91/AZ91 and AZ91–2Ca/AZ91–2Ca welds joined via LFW.

### 3.4. Grain Structure and Texture

**Figure 10:**
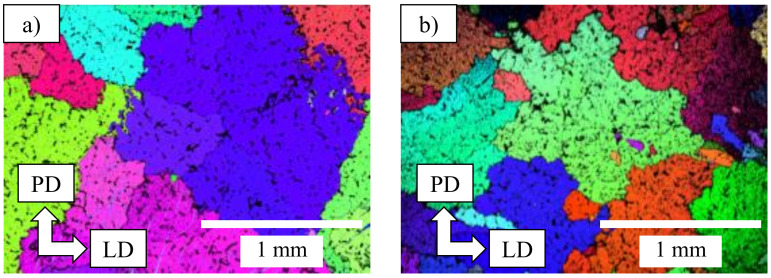
Cast alloys of (**a**) AZ91 and (**b**) AZ91–2Ca before the LFW process. Large grains and thick dendrite arms are observed.

Grain structures and the crystallographic micro-texture of the Mg alloys were analyzed via EBSD. Both cast alloys displayed large grains (>500 µm) with thick dendritic arms (Figure 10). A random texture was observed in both the AZ91 and AZ91–2Ca base materials. This is a common feature observed in metals produced through permanent mold casting.

**Figure 11:**
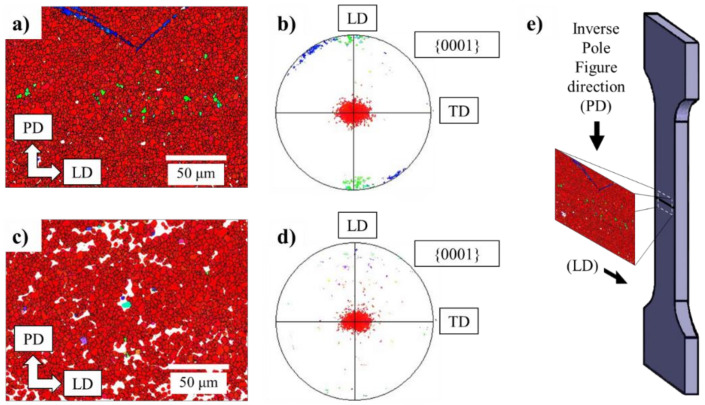
EBSD maps and associated pole figures of the central weld zones in (**a**,**b**) AZ91/AZ91 and (**c**,**d**) AZ91–2Ca/AZ91–2Ca welds, where PD is forging direction, LD is longitudinal direction, and TD is transversal direction. Both weld zones display extensive recrystallization and a strong basal texture perpendicular to the forging direction and (**e**) tensile loading direction.

Figure 11 shows the EBSD maps and pole figures of the central weld zone for both the AZ91/AZ91 and AZ91–2Ca/AZ91–2Ca welds. The inverse pole figure orientations displayed in Figure 11b, d are perpendicular to the forging direction (e.g., Mg basal plane (0001) and parallel to the interface between both coupons. In the central weld zones of both the AZ91/AZ91 and AZ91–2Ca/AZ91–2Ca welds, there is a strong (0001) basal texture (red color-coded grains). Both welds displayed central weld zones with dynamically recrystallized grain structures consisting of fine equiaxed grains that ranged between 2 and 6 µm in size. While the central weld zone of the AZ91–2Ca/AZ91–2Ca weld had a predominantly basal texture, in the AZ91/AZ91 weld, non-basal texture components were also apparent. A few scattered grains had a non-basal {11-20} <10-10> texture component (green color-coded grains) (Figure 11b). There were also a few continuous bands of grains oriented in the non-basal {10-10} <0001> texture component (blue color-coded grains).

### 3.5. Mechanical Properties of the Welded Couples

**Figure 12:**
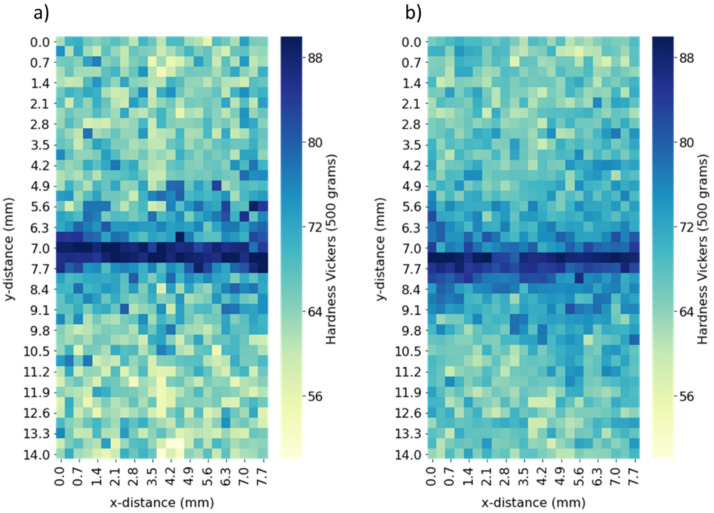
Vickers microhardness maps for the weld region of (**a**) AZ91/AZ91 and (**b**) AZ91–2Ca/AZ91–2Ca welds. As the measurements approached the central weld zone, the hardness increased.

Both Vickers microhardness and static tensile testing were used to gauge the impact of the LFW process on the mechanical properties of the AZ91/AZ91 and AZ91–2Ca/AZ91–2Ca welds. The average Vickers micro-hardness values for the base materials and the central weld zones are shown in Table 3. The Vickers microhardness maps in Figure 12 show that, in both Mg welds, the hardness values tend to increase by up to 32% towards the central weld zone relative to the average values for the AZ91 (65.6 ± 4.0 HV_0.5_) and AZ91–2Ca (66.3 ± 3.1 HV_0.5_) base materials. In addition, the apparent size of the higher hardness weld region in the AZ91/AZ91 weld was observed to be larger than that in the AZ91–2Ca/AZ91–2Ca weld, which is in agreement with microstructural (i.e., grain structure/texture and phase constituent) evolutions, as reported above. However, though the transition from the central weld zone to the base materials was observed microscopically to be rather abrupt, the hardness maps of AZ91/AZ91 and AZ91–2Ca/AZ91–2Ca welds suggest minor hardening in a heat-affected zone (with hardness values ranging from 66 to 87 HV_0.5_) on either side of the central weld zone. In addition, fluctuations in the hardness measurements were noticed, especially in the base material regions, which may be attributed to the presence of large intermetallic particles and casting defects that affect the micro-indentation results.

The mechanical properties of the AZ91/AZ91 and AZ91–2Ca/AZ91–2Ca welds, obtained through tensile testing, are shown in Table 4. The addition of Ca greatly reduced the tensile strength and elongation of AZ91 due to the formation of the fragile Al_2_Ca phase instead of the β–Mg_17_(Al, Zn)_12_ phase, which led to early fracture during tensile loading. The yield strength of the AZ91/AZ91 and AZ91–2Ca/AZ91–2Ca welds was similar or higher than the as-cast base materials (i.e., 15% higher than AZ91–2Ca base material). Tensile fracture of both the AZ91/AZ91 and AZ91–2Ca/AZ91–2Ca welds occurred outside the weld region, close to the edge of the 25 mm gage length region, indicating that the welded region is stronger than the base materials, which agrees with the hardness data in Figure 12.

**Figure 13:**
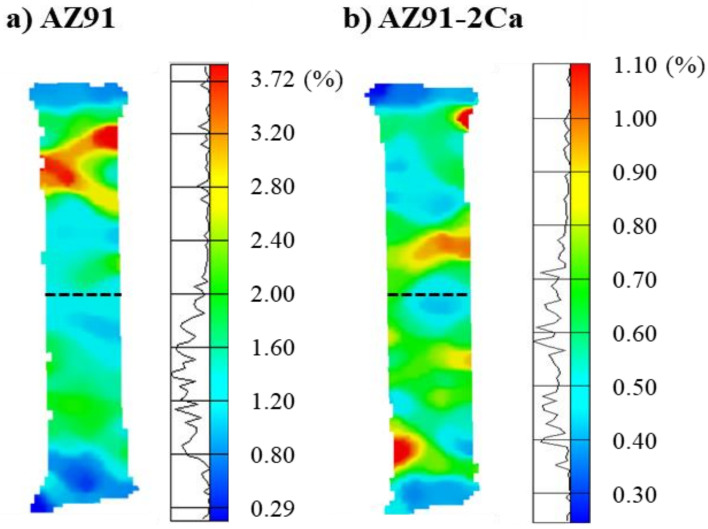
Surface strain map for the (**a**) AZ91/AZ91 and (**b**) AZ91–2Ca/AZ91–2Ca welds right before the final fracture during tensile testing. The black dotted line shows the joint interface. In both welds, fracture occurred in the area of strain localization, outside the weld region (i.e., in the base material regions).

Figure 13 shows the representative surface strain maps for both the AZ91/AZ91 and AZ91–2Ca/AZ91–2Ca welds obtained through digital image correlation with ARAMIS^®^. These maps demonstrate that the highest local strains right before fracture are in the base material region and not within the weld region. In addition, locally, the maximum strain value in the AZ91 base material was 3.7%, which is nearly twice the global strain of 2% for the AZ91/AZ91 weld. Similarly, the maximum value of the local strain in the AZ91–2Ca base material was nearly 1.1%, about 10% higher than the global strain of 1% for the AZ91–2Ca/AZ91–2Ca weld. By contrast, the local strains in the central weld region were considerably lower (~50%), between 1.2 and 1.6% in the AZ91/AZ91 weld and 0.5 and 0.7 in the AZ91–2Ca/AZ91–2Ca weld. This further highlights the higher strength of the central weld region compared to the cast Mg base materials.

## 4. Discussion

### 4.1. Thermal Influences on the Flash and Weld Macroscopic Characteristics

From the thermal camera readings given in Figure 3, LFW of AZ91–2Ca resulted in a higher average temperature than for AZ91. The time elapsed between the maximum/peak temperatures and cooling to 280 °C (lower temperature limit of the camera) was also longer for the AZ91–2Ca relative to AZ91. The solidus temperatures for the AZ91 and AZ91–2Ca alloys are 447 and 472 °C, respectively; thus, these findings point to the processing of the AZ91/AZ91 and AZ91–2Ca/AZ91–2Ca welds in the solid-state condition during LFW.

Previous work has suggested that the ejection of a larger flash from the weld may lead to increased heat dissipation and lower welding temperatures [45]. The stereoscopic images in Figure 4 reveal that the flash thickness formed in the AZ91/AZ91 weld (~4.9 mm) is larger than the flash in AZ91–2Ca/AZ91–2Ca weld (~3.8 mm). This was measured accounting only for the portion of material expelled from the weld body. Hence, the shorter flash produced in AZ91–2Ca/AZ91–2Ca weld is in line with the higher welding temperature measured.

Within the flash, there was also a clear interface between the two sides/coupons of the AZ91/AZ91 and AZ91–2Ca/AZ91–2Ca welds (Figure 4). In particular, both Mg alloys formed a discontinuous flash at the joint interface within the extruded region outside the weld body (Figure 2). From visual inspection, the four sides of the AZ91/AZ91 and AZ91–2Ca/AZ91–2Ca welds did not appear to have formed a continuous flash layer with interconnected edges [46]. However, close examination of the transversely sectioned AZ91/AZ91 and AZ91–2Ca/AZ91–2Ca welds (Figure 4) indicated that the remnant joint interfaces were present only in the flash and ended close to the weld body. In this case, the remnant joint interface defect—that typically acts as a notch and changes the state of stress in a tensile sample [46]—would be removed when machining the flash from the body of the AZ91/AZ91 and AZ91–2Ca/AZ91–2Ca welds. In addition, as mentioned, for the AZ91/AZ91 weld (Figure 2a,b), the flash thickness was ~4.9 mm, but softened material expelled as far as 6 mm from the body of the weld. The AZ91–2Ca/AZ91–2Ca weld (Figure 2c,d), on the other hand, displayed a similar flash thickness of ~3.8 mm, but some material expelled as far as 10 mm away from the welded body. In an AZ31/AA6082 weld, Bhamji et al. [28] reported ample flash being expelled from the AZ31 side but no flash from the aluminum. This is typical of dissimilar alloys welds where preferential material expulsion occurs from the softer alloy with very little (to no) deformation from the stronger alloy during LFW [47,48,49,50]. In the present study, it was interesting to observe the significant material flow during LFW of similar Mg alloys. Though, the high amount of expelled material suggests the opportunity to reduce the process parameters during LFW of AZ91 and AZ91–2Ca, the remnant presence of a joint line in the flash close to the welded body, seems to indicate otherwise. Notwithstanding these extrusion characteristics of the flash, the LFW process conditions applied in the present study realized the formation of a central weld zone that was continuously bonded across the joint (from edge-to-edge) and had a small thickness of around 1.5 mm in the AZ91/AZ91 weld and 0.9 mm in the AZ91–2Ca/AZ91–2Ca weld. The size of this central weld zone is similar to that reported for LFW of other lightweight alloys, such as near-α, α + β, and near-β titanium alloys [51,52,53,54].

### 4.2. Microstructure and Texture

*Microstructures:* From the thermal history during LFW of the Mg alloys, the weld regions reached average temperatures of 351 °C (AZ91/AZ91) and 407 °C (AZ91–2Ca/AZ91–2Ca). The room temperature microstructures in the weld region after LFW are expected to result from rapid cooling (quenching) of the phase constituents existing at these elevated temperatures. Table 4 and Table 5 indicate the phases present in the two Mg alloys, AZ91 and AZ91–2Ca, respectively, at their peak temperatures calculated through FactSage-FTlite™. To aid the discussions, the phases predicted in the as-cast structures of the base materials—as given in Table 2—are also shown in Table 5 and Table 6 for the AZ91/AZ91 and AZ91–2Ca/AZ91–2Ca welds, respectively.

*AZ91/AZ91 weld:* The simulations indicate that the amount of the β–Mg_17_Al_12_ phase in the AZ91 alloy reduces from the room temperature value of ~11.9 wt% in an as-cast structure to 3.3 wt% in the weld region at the maximum average temperature of 351 °C recorded during LFW (Table 5). In addition, the Al_8_Mn_5_ phase in the AZ91 at room temperature transforms into Al_4_Mn at 351 °C. A comparison of the simulated results for the amount of the Al–Mn phase in weld region of the AZ91 alloy showed a higher (double) fraction at 351 °C relative to that in an as-cast AZ91 structure at room temperature.

*AZ91–2Ca/AZ91–2Ca weld:* For the AZ91–2Ca/AZ91–2Ca weld simulation, no β–Mg_17_Al_12_ phase particles remain at the maximum average temperature of 407 °C recorded during LFW. This prediction agrees with the absence of the β–Mg_17_Al_12_ phase particles in the microscopic/EDS observations of AZ91–2Ca/AZ91–2Ca weld region (Table 6), as well as with the measured results from the XRD analysis, as shown in Figure 5b. The amount of Al_2_Ca particles remains unchanged (as it only disperses) at ~4.6 wt%, which also agrees with the as-welded microstructures given in Figure 8 and Figure 9. In addition, phase transformations are predicted in the Mn–Al phases. Specifically, thermodynamic calculations indicate that the Al_8_Mn_5_ particles dissolve in the AZ91–2Ca/AZ91–2Ca weld and, instead, the ternary Mn_2_CaAl_10_ phase is present. This phase transformation is the only feature that does not completely agree with the observed EDS analysis, as the Al–Mn phases seem to contain no Ca. This can be attributed to the EDS detection limit or an incomplete transformation due to the short time elapsed at the high temperatures during LFW.

*High Temperature Deformation, Recrystallization, and Texture*: Both Mg alloys exhibit strong basal recrystallization textures after LFW. The basal planes rotate perpendicular to the compression axis (in this case, the forging direction) during deformation. The most common recrystallization mechanism that is seen in Mg alloys is related to grain-boundary bulging that will yield recrystallization textures similar to the texture of parent grains, which are basal. It is reported that in FSW, a strong basal texture is generated perpendicular to the force direction to accommodate the deformation induced by the rotative pin [55]; we can conclude that both Mg alloys during LFW have behaved similarly to FSW. Interestingly, the AZ91 alloy exhibits minor amounts of non-basal texture components (Figure 11b): (i) Bands of grains oriented in the prismatic {10-10} <0001> texture component and (ii) a few scattered grains with a {11-20} <10-10> texture component. These components have been observed in rolling textures of AZ31 at high temperatures. The homologous temperature, T_H_, at the respective weld peak-temperatures of AZ91 and AZ91–2Ca are 0.87 and 0.91, respectively. Despite the higher weld-peak and homologous temperatures, AZ91–2Ca does not exhibit non-basal texture components activated by high temperature deformation. It can be inferred that the mechanism for the generation of the non-basal grains in AZ91 is other than grain boundary bulging. Other mechanisms of non-basal texture in recrystallized grains of Mg alloys may be due to particle-stimulated nucleation (PSN) or twin-induced nucleation (TIN).

*Particle-Stimulated Nucleation (PSN)*: The microstructural investigation and the thermodynamic simulations in this work show that while the β–Mg_17_(Al, Zn)_12_ s phase particles disappeared and the Al_2_Ca phase remained in the AZ91–2Ca weld, very little second phase (β–Mg_17_(Al, Zn)_12_) remained in the AZ91 weld. In Mg–Al-based alloys, the β–Mg_17_(Al, Zn)_12_ phase has not been associated with the nucleation of non-basal grain orientations, hence, it is unlikely that the non-basal texture component is a result of PSN. Since AZ91–2Ca weld does not show any non-basal texture component, the Ca phase does not seem to have a role in texture weakening either.

*Twin-Induced Nucleation (TIN)*: In this study, non-basal texture was observed in the shear bands of the AZ91 weld. Shear-bands in Mg occur as a result of discontinuous recrystallization (grain boundary bulging) or twinning [56], especially under compression. Shear bands usually undergo subsequent twinning as well. The propensity to twinning and the twinning modes are highly alloy dependent through the c/a ratio in HCP materials [57]. As an example, the {10-12} twins are commonly seen in high temperature rolling of AZ31 [58]. Twinning induced dynamic recrystallization has also been confirmed in AZ61 alloy under deformation at 450 °C [59]. It can be deduced that non-basal components in AZ91/AZ91 linear friction welds are likely twin induced. Since twinning is enhanced by larger grain sizes [60], it can also be deduced that twinning was less significant in the AZ91–2Ca alloy weld due to the smaller as-cast grain size of this alloy compared to AZ91 (Figure 11).

### 4.3. Mechanical Properties

*Base alloys:* AZ91–2Ca shows a lower yield strength, ultimate tensile strength, and ductility compared to AZ91. The lower ductility in the AZ91–2Ca base material compared to AZ91 is due to the additional brittle secondary phase, Al_2_Ca, which also accounts for the lower ultimate tensile strength and final strain (EL) values reached. AZ91–2Ca also shows a lower yield strength due to the lower amount of the β–Mg_17_(Al, Zn)_12_ phase.

*AZ91/AZ91 and AZ91–2Ca/AZ91–2Ca welds:* Tensile fracture of the welds for both Mg alloys occurred outside the weld region. This is readily explained by the recrystallized basal texture seen in the central weld zone of the AZ91/AZ91 and AZ91–2Ca/AZ91–2Ca welds. The basal planes of the central welded zone are oriented perpendicular to the tensile axis (Figure 11e) giving a very low Schmidt factor for slip and a higher local yield strength for the central weld zone relative to the base materials. This is also supported by the hardness data. The overall (global) tensile properties of the welds have contributions from the base material region and the weld zone. Thus, the strength of the weld zone contributes to the higher yield strength of the AZ91/AZ91 and AZ91–2Ca/AZ91–2Ca welds compared to the (un-welded) base materials. It is important to note that no significant increase in local elongation (strain) was observed in the vicinity of the weld zone for both the AZ91/AZ91 and AZ91–2Ca/AZ91–2Ca welds.

## 5. Conclusions

Developing the LFW process for joining Mg alloys is of interest for assembly of net-shape parts with complex geometries that cannot be joined through other advanced methods, such as friction stir welding. The present research is a first study (to the best knowledge of the authors) on evaluating the feasibility of applying LFW to join AZ91 to AZ91 and AZ91–2Ca to AZ91–2Ca. The following conclusions can be drawn based on the observations of the microstructural phase constituents, texture, microhardness evolution, and tensile properties of the AZ91/AZ91 and AZ91–2Ca/AZ91–2Ca welds:

1. For the parametric conditions examined for LFW, the AZ91/AZ91 and AZ91–2Ca/AZ91–2Ca welds exhibited intimate bonding without the presence of discontinuities such as pores, voids, and/or cracks at the joint interface.
2. Both alloys showed microstructural changes with increasing temperature during LFW. At the welding temperatures, the β–Mg_17_(Al, Zn)_12_ phase dissolved, enriching the α–Mg matrix in Al. The Al_8_Mn_5_ phase remained in both Mg alloys and the Al_2_Ca remained in AZ91–2Ca, albeit evenly dispersed through the central weld zone due to deformation.
3. The LFW process generated a recrystallized microstructure in the central weld zone of the AZ91/AZ91 and AZ91–2Ca/AZ91–2Ca welds. Compared to large grain size of AZ91 and AZ91–2Ca base materials (~1000 µm), the fine equiaxed grains in the central weld zone were about 2–6 µm in size. This grain refinement contributed to increase in the hardness of the central weld zone to 87 ± 3.0 HV_0.5_ and 88 ± 2.7 HV_0.5_ in the AZ91/AZ91 and AZ91–2Ca/AZ91–2Ca welds, respectively. Relative to the AZ91 and AZ91–2Ca base material, the central weld zone was about 32% harder.
4. Tensile testing of the AZ91/AZ91 and AZ91–2Ca/AZ91–2Ca welds was undertaken using digital image correlation to measure the strain distribution in the gauge length section of the tensile samples. The tensile strength properties of the welds were similar or slightly higher than the base materials and final failure occurred exclusively in the AZ91 and AZ91–2Ca base materials. Examination of the strain distribution across the AZ91/AZ91 and AZ91–2Ca/AZ91–2Ca joints just before final fracture indicated that the local strains in the central weld zone were about 50% lower than those in the base material regions.
5. A basal recrystallization texture developed in the weld region of the AZ91/AZ91 and AZ91–2Ca/AZ91–2Ca joints, such that the basal planes were oriented perpendicular to the LFW forging direction and the tensile stress axis. This strong basal texture also strengthened the weld region locally (as evidenced by the lower local strains in the central weld zone) relative to the base material during tensile testing.

## Figures and Tables

**Table 1:** Chemical Composition of the AZ91–2Ca alloy (wt.%).

Element	Al	Ca	Si	Cu	Fe	Mn	Ni	Zn	Mg
AZ91–2Ca	9.71	2.23	–	<0.001	0.009	0.34	<0.001	1.13	Balance
AZ91 [35]	9.64	–	0.03	0.01	0.004	0.31	<0.001	0.78	Balance

**Table 2:** Phase prediction in as-cast alloys at room temperature using FactSage-FTlite™ thermodynamic calculations. Calculated phases with a mass fraction below 0.1 wt% are not displayed.

Alloy	Phase-Crystal Structure	Phase wt.%
**AZ91**	α Mg–HCP–A3	87.44
	β–Mg_17_(Al, Zn)_12_	11.92
	Al_8_Mn_5_–D8_10_	0.38
**AZ91–2Ca**	α Mg–HCP–A3	87.65
	β–Mg_17_(Al, Zn)_12_	6.68
	Al_8_Mn_5_–D8_10_	0.28
	Al_2_Ca–C_15_	4.57
	Mg_6_(Al,Zn)_5_–φ	0.37

**Table 3:** Average Vickers microhardness values for the base materials and the central weld zone in both welds.

Weld	Average Hardness (HV_0.5_)	
	Base Material	Central Weld Zone
AZ91/AZ91	65.6 ± 4.0	87 ± 3.0
AZ91–2Ca/AZ01–2Ca	66.3 ± 3.1	88 ± 2.7

**Table 4:** Mechanical properties for both cast and welded alloys.

Materials	UTS (MPa)	YS (MPa)	Final Strain or EL (%)
LFW AZ91	138	95	2
LFW AZ91–2Ca	99	86	1
AZ91 [35]	153	93	2
AZ91–2Ca	110	75	1

**Table 5:** Phase predictions for AZ91 at the maximum welding temperature and in the as-cast state.

AZ91 Welded Structure (Quenched from 351 °C)		AZ91 As-Cast Structure	
Phases	Amount of Phase, wt%	Phases	Amount of Phase, wt%
α Mg–HCP–A3	95.85	α Mg–HCP–A3	87.44
β–Mg_17_(Al, Zn)_12_	3.26	β–Mg_17_(Al, Zn)_12_	11.92
Al_4_Mn	0.89	Al_8_Mn_5_–D8_10_	0.38

**Table 6:** Phase predictions for AZ91–2Ca at the maximum welding temperature and in the as-cast state.

AZ91–2Ca Welded Structure (Quenched from 407 °C)		AZ91–2Ca As-Cast Structure	
Phases	Amount of Phase, wt%	Phases	Amount of Phase, wt%
α Mg–HCP–A3	94.24	α Mg–HCP–A3	87.65
β–Mg_17_(Al, Zn)_12_	0	β–Mg_17_(Al, Zn)_12_	6.68
Mn_2_CaAl_10_	1.1461	Al_8_Mn_5_–D8_10_	0.28
Al_2_Ca–C_15_	4.61	Al_2_Ca–C_15_	4.57

## Data Availability

The authors confirm that the data supporting the findings of this study are available within the article.

## Abbreviations

AA	Aluminum alloy
Al	Aluminum
Ar	Argon
ASTM	American Society for Testing and Materials
AZ	Magnesium, Aluminum, Zinc, and Manganese alloys
BSE	Backscattered Electron
Ca	Calcium
D	Depth
EBSD	Electron Backscattered Diffraction
EL	Elongation
FSP	Friction Stir Processing
FSW	Friction Stir Welding
HCP	Hexagonal Close-Packed
He	Helium
HV	Hardness Vickers
ICP-AES	Inductively Coupled Plasma-Atomic Emission Spectrometry
L	Length
LFW	Linear Friction Welding
Mg	Magnesium
NADCAP	National Aerospace and Defense Contractors Accreditation Program
NIST	National Institute of Standards and Technology
PDS	Process Development System
PSN	Particle Stimulated Nucleation
SEM	Scanning Electron Microscope
TIN	Twin Induced Nucleation
UTS	Ultimate Tensile Strength
W	Width
XRD	X-Ray Diffraction
YS	Yield Strength
Zn	Zinc
ρ	Density
